# Supplementary material for: Splice-Junction-Based Mapping of Alternative Isoforms in the Human Proteome
Source: Cell Rep. Author manuscript; Available in PMC 2020 Jan 15. (PMC6961840; doi:10.1016/j.celrep.2019.11.026)
Supplement: 2 [file NIHMS1546469-supplement-2.zip › DF1/PXD009737/TestisGluC/TestisGluC_16_TMEM94_KQLPPPQEPPSALGLSTRKA.pdf]

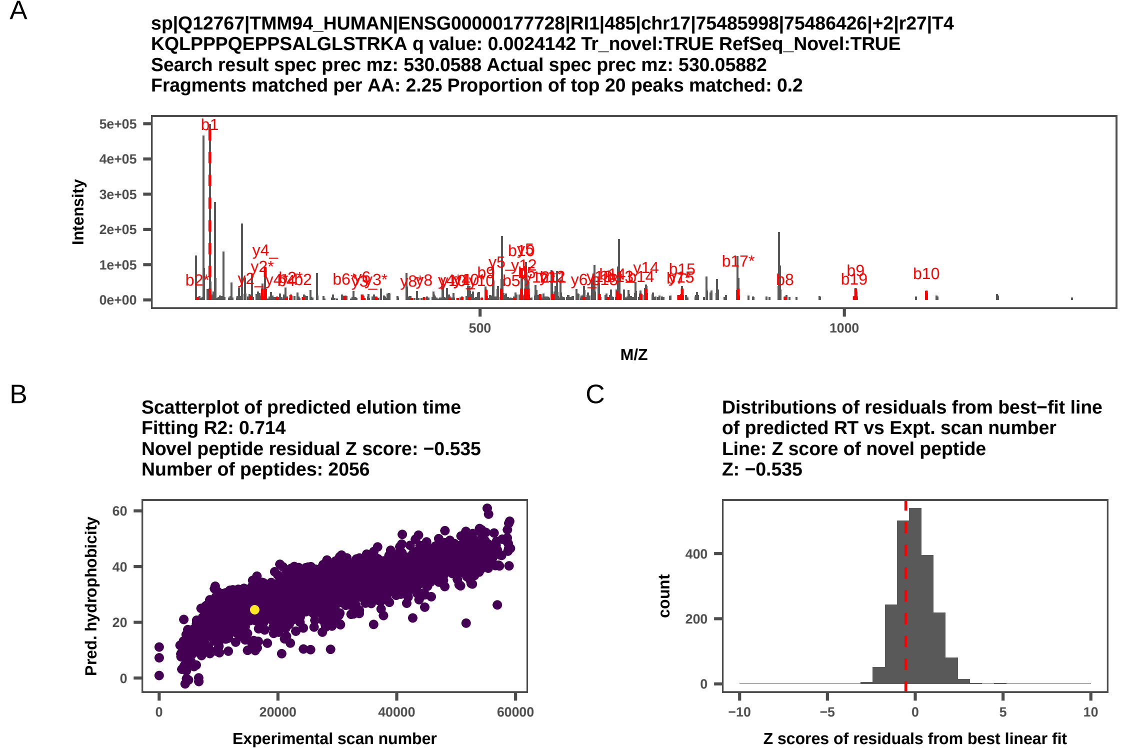
A
sp|Q12767|TMM94_HUMAN|ENSG00000177728|RI1|485|chr17|75485998|75486426|+2|r27|T4
KQLPPPQEPPSALGLSTRKA q value: 0.0024142 Tr_novel:TRUE RefSeq_Novel:TRUE
Search result spec prec mz: 530.0588 Actual spec prec mz: 530.05882
Fragments matched per AA: 2.25 Proportion of top 20 peaks matched: 0.2
Intensity
M/Z
B
Scatterplot of predicted elution time
Fitting R2: 0.714
Novel peptide residual Z score: −0.535
Number of peptides: 2056
Pred. hydrophobicity
Experimental scan number
C
Distributions of residuals from best−fit line
of predicted RT vs Expt. scan number
Line: Z score of novel peptide
Z: −0.535
count
Z scores of residuals from best linear fit
